# Thermodynamic Limit of Electroluminescent Refrigeration Devices

**DOI:** 10.3390/e27050496

**Published:** 2025-05-04

**Authors:** A. N. M. Fuhadul Islam, S. Mostafa Ghiaasiaan, Zhuomin M. Zhang

**Affiliations:** George W. Woodruff School of Mechanical Engineering, Georgia Institute of Technology, Atlanta, GA 30332, USA; aislam81@gatech.edu (A.N.M.F.I.);

**Keywords:** detailed balance model, electroluminescence, endoreversible thermodynamics, entropy analysis, solid-state refrigeration

## Abstract

This work explores the thermodynamic performance limit of electroluminescent refrigeration systems using entropy analysis. A typical electroluminescent (EL) refrigeration device cools a contacting object by emitting radiation to a high-temperature receiver. Conversely, a negative electroluminescent (NEL) refrigeration device cools a low-temperature target by absorbing radiation from it. This study performs a second-law analysis of these systems using the ideal diode model based on endoreversible thermodynamics. The results are compared with those from the detailed balance model for refrigeration systems composed of diodes of various bandgaps. It is found that entropy analysis consistently predicts better cooling performance than the detailed balance model. Additionally, this study examines the impact of the temperature difference between the emitter and the receiver in both EL and NEL refrigeration systems. The insights from this study set a theoretical benchmark for electroluminescent refrigeration systems for maximum cooling performance.

## 1. Introduction

Biasing a semiconductor diode results in splitting the quasi-Fermi levels of electrons and holes, causing the emitted photons to possess a chemical potential [1,2]. The development of the photon chemical potential modifies the conventional Planck’s law of blackbody radiation distribution. A positive photon chemical potential is built under forward bias, and vice versa. For photon energies greater than the semiconductor bandgap, a positive chemical potential increases the emitted photon flux, whereas a negative chemical potential reduces the emitted photon flux [3]. This phenomenon is harnessed in electroluminescent (EL) and negative electroluminescent (NEL) refrigeration systems. These devices have attracted attention because of their solid-state nature and potential for higher efficiencies compared to existing solid-state thermoelectric cooling. In an EL refrigeration system, a cold *p*-*n* junction is forward-biased and emits net radiation to a hot receiver. This facilitates heat transfer from a solid object in contact with the diode, enabling the object to be maintained at a lower temperature by consuming electrical power. The process is just like a conventional refrigeration cycle. Likewise, in an NEL system, a reverse bias is applied to a semiconductor diode, which behaves as a heat sink that can absorb radiative energy from a target that is maintained at a lower temperature to create a refrigeration effect.

The concept of EL cooling was proposed in the 1950s [4,5] and first demonstrated by Dousmanis et al. [6] using GaAs diodes in 1964. Weinstein [7] performed a thermodynamic analysis to study the limit of the heat-to-light conversion efficiency, showing that it could reach 160% in EL devices operating near room temperature. Berdahl [8] analyzed the cooling power density and coefficient of performance (*COP*) of NEL refrigeration systems. Subsequently, Berdahl et al. [9] summarized the NEL measurements of several narrow band materials and demonstrated a cooling effect with InSb in their calorimetric experiment. Due to the internal nonradiative losses and sub-bandgap thermal radiation, the poor cooling performance has largely limited the use of EL and NEL for refrigeration purposes, despite the success of studying EL in light-emitting devices (LEDs) across the spectra.

The continuous development of semiconductor materials and the progress in using selective emitters and near-field radiation have revived the interest in using the EL and NEL phenomena for noncontact refrigeration applications [2,3,10,11]. Santhanam et al. [12,13] demonstrated EL cooling of mid-infrared LEDs with an electrical-to-optical conversion efficiency exceeding 100%, resulting in a pumping of heat from the crystal lattice. Piprek and Li [14] illustrated a cooling effect with wide bandgap InGaN/GaN blue LEDs. Chen et al. [15,16], Liu and Zhang [17], and Lin et al. [18] theoretically calculated a large cooling power density enabled by near-field radiation effect that can exceed the far-field limit by 2–3 orders of magnitude. Subsequently, near-field EL cooling was experimentally demonstrated by Zhu et al. [19] with an InAsSb photodiode. The architecture of combining EL cooling with a photovoltaic generator, also called a thermophotonic heat pump, has also been investigated to enhance the cooling performance [20,21,22,23]. Furthermore, intracavity double diode structures have been used to examine the EL cooling performance by integrating a photodetector to directly measure LED efficiency [24,25,26]. Multijunction EL and NEL systems have also been recently analyzed [27] based on a detailed balance model [28]. Electroluminescent refrigeration is advantageous in cryogenic cooling since the quantum efficiency can significantly improve at low operating temperatures [22]. EL and NEL cooling may also be used in solid-state cooling devices for space applications and thermal management due to their compactness and absence of any moving parts [14,15,16,17,18,19,20,21,22,23]. Despite the progress made in the theoretical and experimental studies of electroluminescent refrigeration, an entropy or second-law analysis to establish a fundamental thermodynamic limit of cooling performance of these systems has remained underexplored.

Thermal radiation carries both energy and entropy [12]. Studies have shown that the relation between radiation energy and entropy is not straightforward like conduction [29,30]. While entropy analysis has been used, both in the past [7] and more recently [31], to determine the thermodynamic bound for the efficiency of LEDs, these studies rely on an overly simplified model of photon entropy without considering photon chemical potential. Research on the thermodynamic limit of different radiative thermoelectric converters, such as photovoltaic [32] and thermoradiative cells [33,34,35,36], using entropy analysis has been performed. Li et al. [37] performed an entropy analysis to determine the thermodynamic performance of near-field EL and NEL refrigeration systems, though their method for evaluating near-field entropy flux is still controversial [38]. The present study aims to establish a fundamental limit of maximum cooling power density and *COP* of EL and NEL refrigeration systems in the far field across various bandgaps of semiconductors. Both refrigeration systems are analyzed using the second law of thermodynamics and detailed balance analysis. Calculated results from these two methods are then compared. Finally, the impact of the temperature difference between the emitter and receiver is explored in terms of refrigeration performance.

## 2. Theoretical Background

### 2.1. Geometrical Configuration

**Figure 1:**
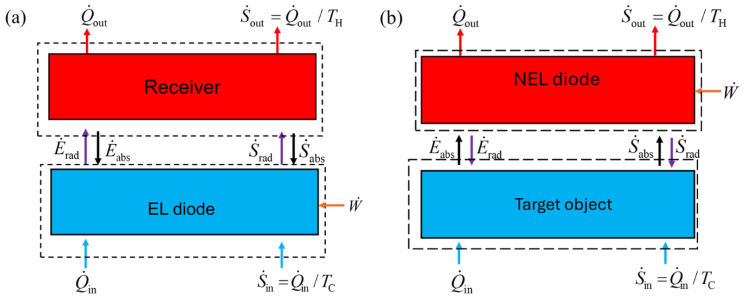
Schematic of energy and entropy balances of (**a**) the EL refrigeration system and (**b**) the NEL refrigeration system. The dotted boxes represent the control volume of the diodes and the corresponding receiver and the target object. Here, ${\dot{E}}_{\mathrm{rad}}$ (or ${\dot{S}}_{\mathrm{rad}}$) and ${\dot{E}}_{\mathrm{abs}}$ (or ${\dot{S}}_{\mathrm{abs}}$) denote the emitter and absorbed radiative energy (or entropy) fluxes by the diode, respectively.

An EL refrigeration system consists of an EL diode (or emitter) and a receiver, as shown in Figure 1a. Heat and entropy are conducted to the diode from a cold region (that is to be refrigerated) in contact with the diode. The heat and entropy transfer rates are denoted as ${\dot{Q}}_{\mathrm{in}}$ and ${\dot{S}}_{\mathrm{in}}$, respectively. Under the steady-state condition, the diode is maintained at a temperature of *T*_C_. When a forward bias is applied to the *p*-*n* junction, the diode emits a net number of photons, which are taken in by the receiver that is kept at *T*_H_ and transfers heat (at a rate of ${\dot{Q}}_{\mathrm{out}}$) and entropy (at a rate of ${\dot{S}}_{\mathrm{out}}$) to a higher-temperature reservoir via heat conduction. The second law of thermodynamics imposes a requirement that work or power ($\dot{W}$) must be provided to cause heat to flow from cold to hot.

As shown in Figure 1b, an NEL refrigeration system consists of an emitter (target object) and a receiver denoted as NEL diode, which is a *p*-*n* junction under reverse bias. Despite being at a higher temperature of *T*_H_, the NEL diode absorbs thermal radiation from the cooler target object that is kept at a lower temperature of *T*_C_. Heat and entropy are conducted to the emitter from a cold reservoir and transferred from the receiver to a hot reservoir via conduction. Electric power $\dot{W}$ is provided to the diode during the process to drive the refrigeration effect. In both EL and NEL systems, ${\dot{Q}}_{\mathrm{in}}$ is the cooling load. All the energy and entropy terms shown in Figure 1 are normalized to the area of the diode.

### 2.2. Second-Law Analysis

To assess the limiting performance, it is assumed that the semiconductor diodes are ideal and follow the detailed balance formulation [28] without any losses due to nonradiative processes and lattice or surface imperfections. Furthermore, the emissivity of the diode surfaces is assumed to be unity for photon energies greater than the bandgap (*E*_g_), and zero elsewhere. The surfaces facing the diode are treated as blackbodies. A single photon taken in or emitted by the semiconductor *p*-*n* junction corresponds to a generation or recombination of an electron-hole pair. Throughout the semiconductor diode, the developed photon chemical potential (μ) is constant and proportional to the bias voltage (*V*), i.e., μ=qV, where *q* is the elementary charge. Based on these assumptions, the spectral energy flux of the diode can be calculated by [36]:

(1) $${\dot{E}}_{\omega }(\omega ,T,\mu )=\left\{\begin{array}{c} \frac{\hbar \omega^{3}}{4\pi^{2}c^{2}}f_{\mathrm{BE}}\left(\omega ,T,\mu \right),\omega >\omega_{g}\\ 0,\omega <\omega_{g} \end{array}\right.$$

Here, *ω* is the angular frequency, ℏ is the reduced Planck constant, *c* is the speed of light in a vacuum, $\omega_{g}=E_{g}/\hbar$ is the frequency corresponding to the bandgap, and *f*_BE_ is the modified Bose-Einstein distribution function that can be expressed as [2,3,27,36]:

(2) $$f_{\mathrm{BE}}\left(\omega ,T,\mu \right)=\frac{1}{\exp\left(\frac{\hbar \omega -\mu }{k_{B}T}\right)-1}$$

where *k*_B_ is the Boltzmann constant. As mentioned before, *µ* is positive for an EL system and negative for an NEL system. Note that Equations (1) and (2) can also be applied to blackbody surfaces by setting μ=0 and $\omega_{g}=0$.

In an EL refrigeration system, the net radiative energy transfer (or flux) out from the diode can be calculated by:

(3) $${\dot{E}}_{\mathrm{net},\mathrm{EL}}={\dot{E}}_{\mathrm{rad}}-{\dot{E}}_{\mathrm{abs}}=\int_{\omega_{g}}^{\omega_{\mathrm{cut},\mathrm{EL}}}\left[{\dot{E}}_{\omega }\left(\omega ,T_{C},\mu \right)-{\dot{E}}_{\omega }\left(\omega ,T_{H},0\right)\right]d\omega$$

An integration upper limit or cut-off frequency $\omega_{\mathrm{cut},\mathrm{EL}}$ is set in Equation (3), and this will be explained later. The steady-state energy balance of the EL diode yields:

(4) $${\dot{E}}_{\mathrm{net},\mathrm{EL}}={\dot{Q}}_{\mathrm{in}}+\dot{W}$$

In an NEL refrigeration system, there is a net absorption (${\dot{E}}_{\mathrm{net},\mathrm{NEL}}$) of photon energy by the NEL diode from the target object. The absorbed photon energy flux, along with the input power ($\dot{W}$), is transferred to the hot reservoir via conductive heat transfer, as shown in Figure 1b. The energy balance for the NEL diode gives:

(5) $${\dot{Q}}_{\mathrm{out}}={\dot{E}}_{\mathrm{net},\mathrm{NEL}}+\dot{W}=\int_{\omega_{g}}^{\omega_{\mathrm{cut},\mathrm{NEL}}}\left[{\dot{E}}_{\omega }\left(\omega ,T_{C},0\right)-{\dot{E}}_{\omega }\left(\omega ,T_{H},\mu \right)\right]d\omega +\dot{W}$$

where ${\dot{E}}_{\mathrm{net},\mathrm{NEL}}={\dot{Q}}_{\mathrm{in}}$ is the cooling load. In Equations (3) and (5), the lower bound is *ω*_g_, according to Equation (1). For effective cooling to take place, an upper bound frequency is imposed for either EL or NEL, because the integrand becomes negative at higher frequencies. The cut-off frequencies are determined by [2,8,27]:

(6) $$\omega_{\mathrm{cut},\mathrm{EL}}=\frac{qV/\hbar }{1-T_{C}/T_{H}}\;\mathrm{and}\;\omega_{\mathrm{cut},\mathrm{NEL}}=\frac{(-qV)/\hbar }{T_{H}/T_{C}-1}$$

The upper bound frequency is used to determine the optimal theoretical performance. In practice, a low-pass optical filter needs to be placed between the emitter and the receiver to reflect (block) the emission at frequencies exceeding the cut-off frequency. This is especially important when the absolute bias voltage is low.

For effective cooling to occur, the bias voltage must fall into a certain range. For the EL system, this requires that *V*_th,EL_ ≤ *V* < *E*_g_/*q*, and, for the NEL system, *V* < *V*_th,NEL_. The threshold voltages for EL and NEL are, respectively [2,8]:

(7) $$V_{\mathrm{th},\mathrm{EL}}=\frac{E_{g}}{q}\left(1-\frac{T_{C}}{T_{H}}\right)\;\mathrm{and}\;V_{\mathrm{th},\mathrm{NEL}}=-\frac{E_{g}}{q}\left(\frac{T_{H}-T_{C}}{T_{C}}\right)$$

It should be noted that Equation (7) is a consequence of Equation (6), since the threshold voltage corresponds to the voltage that gives a cut-off frequency equal to $\omega_{g}$.

The photon flux is not only accompanied by an energy flux but also by an entropy flux. Based on statistical thermodynamics, the spectral entropy flux is expressed as [1,3,11,36]:

(8) $${\dot{S}}_{\omega }(\omega ,T,\mu )=\frac{k_{B}\omega^{2}}{4\pi^{2}c^{2}}\left[\left(1+f_{\mathrm{BE}})\ln(1+f_{\mathrm{BE}})-f_{\mathrm{BE}}\ln(f_{\mathrm{BE}}\right)\right]$$

The net entropy flux from the EL diode can be calculated by:

(9) $${\dot{S}}_{\mathrm{net},\mathrm{EL}}={\dot{S}}_{\mathrm{rad}}-{\dot{S}}_{\mathrm{abs}}=\int_{\omega_{g}}^{\omega_{\mathrm{cut},\mathrm{EL}}}\left[{\dot{S}}_{\omega }(\omega ,T_{C},\mu )-{\dot{S}}_{\omega }(\omega ,T_{H},0)\right]d\omega$$

Similarly, the net entropy flux to the NEL diode can be calculated by:

(10) $${\dot{S}}_{\mathrm{net},\mathrm{NEL}}={\dot{S}}_{\mathrm{abs}}-{\dot{S}}_{\mathrm{rad}}=\int_{\omega_{g}}^{\omega_{\mathrm{cut},\mathrm{NEL}}}\left[{\dot{S}}_{\omega }(\omega ,T_{C},0)-{\dot{S}}_{\omega }(\omega ,T_{H},\mu )\right]d\omega$$

The entropy balances may be applied to the EL refrigeration system considering the two control volumes given in Figure 1a for both the diode and the receiver. The receiver absorbs entropy from the photons at the rate of ${\dot{S}}_{\mathrm{net},\mathrm{EL}}$ and transfers entropy through conduction to the hot reservoir at the rate of ${\dot{S}}_{\mathrm{out}}={\dot{Q}}_{\mathrm{out}}/T_{H}={\dot{E}}_{\mathrm{net},\mathrm{EL}}/T_{H}$. The entropy generated rate per unit area of the receiver is, therefore:

(11) $${\dot{\sigma }}_{H,\mathrm{EL}}=\frac{{\dot{E}}_{\mathrm{net},\mathrm{EL}}}{T_{H}}-{\dot{S}}_{\mathrm{net},\mathrm{EL}}$$

Because of Equation (11), an EL refrigeration system is inherently irreversible. For specified reservoir temperatures and bandgap energy of the semiconductor, the entropy generated in the receiver only depends on the photon chemical potential (or bias voltage). For the diode, the entropy balance can be established by considering the heat conduction from the cold reservoir and net outgoing entropy of photons. Hence, the entropy generation rate per unit area of the diode is:

(12) $${\dot{\sigma }}_{C,\mathrm{EL}}={\dot{S}}_{\mathrm{net},\mathrm{EL}}-{\dot{S}}_{\mathrm{in}}$$

The best performance of the refrigeration system is achieved when the endoreversible condition is adopted for the diode, i.e., ${\dot{\sigma }}_{C,\mathrm{EL}}$ = 0 [36]. The expressions of the maximum cooling load, input electric power, and coefficient of performance (*COP*) can be respectively expressed as follows:

(13) $${\dot{Q}}_{\mathrm{in},2\mathrm{nd},\mathrm{EL}}=T_{C}{\dot{S}}_{\mathrm{in},2\mathrm{nd},\mathrm{EL}}=T_{C}{\dot{S}}_{\mathrm{net},\mathrm{EL}}$$

(14) $${\dot{W}}_{2\mathrm{nd},\mathrm{EL}}={\dot{E}}_{\mathrm{net},\mathrm{EL}}-T_{C}{\dot{S}}_{\mathrm{net},\mathrm{EL}}$$

and

(15) $$COP_{2\mathrm{nd},\mathrm{EL}}=\frac{{\dot{Q}}_{\mathrm{in},2\mathrm{nd},\mathrm{EL}}}{{\dot{W}}_{2\mathrm{nd},\mathrm{EL}}}=\frac{{\dot{S}}_{\mathrm{net},\mathrm{EL}}}{{\dot{E}}_{\mathrm{net},\mathrm{EL}}/T_{C}-{\dot{S}}_{\mathrm{net},\mathrm{EL}}}$$

The subscript “2nd” signifies that the corresponding quantity is obtained from the second-law analysis under the endoreversible assumption. Equation (15) shows that the second-law analysis sets up a performance limit that does not rely on the diode behavior, except for the bandgap of the semiconductor and the bias voltage that is needed in the modified Bose-Einstein distribution.

Entropy balance can also be applied to the emitter and receiver of the NEL shown in Figure 1b. This system is also inherently irreversible as entropy generation in the target object cannot be eliminated. The maximum cooling load and *COP* can be obtained using the endoreversible condition for the diode: ${\dot{\sigma }}_{H,\mathrm{NEL}}={\dot{Q}}_{\mathrm{out},2\mathrm{nd},\mathrm{NEL}}/T_{H}-{\dot{S}}_{\mathrm{net},\mathrm{NEL}}=0$. Subsequently, the cooling load, power input, and *COP* of an NEL refrigeration system can be expressed as follows, according to the second-law analysis:

(16) $${\dot{Q}}_{\mathrm{in},2\mathrm{nd},\mathrm{NEL}}={\dot{E}}_{\mathrm{net},\mathrm{NEL}}$$

(17) $${\dot{W}}_{2\mathrm{nd},\mathrm{NEL}}={\dot{Q}}_{\mathrm{out},2\mathrm{nd},\mathrm{NEL}}-{\dot{E}}_{\mathrm{net},\mathrm{NEL}}=T_{H}{\dot{S}}_{\mathrm{net},\mathrm{NEL}}-{\dot{E}}_{\mathrm{net},\mathrm{NEL}}$$

and

(18) $$COP_{2\mathrm{nd},\mathrm{NEL}}=\frac{{\dot{Q}}_{\mathrm{in},2\mathrm{nd},\mathrm{NEL}}}{{\dot{W}}_{2\mathrm{nd},\mathrm{NEL}}}=\frac{{\dot{E}}_{\mathrm{net},\mathrm{NEL}}}{T_{H}{\dot{S}}_{\mathrm{net},\mathrm{NEL}}-{\dot{E}}_{\mathrm{net},\mathrm{NEL}}}$$

Once the temperatures of the emitter and receiver and bandgap of the semiconductor are prescribed, the bias voltage (or chemical potential) is the only parameter that is needed to determine the cooling load and *COP* for both the EL and NEL systems.

### 2.3. Detailed Balance Model

Similar to Equation (1), the spectral photon flux is given by [36]:

(19) $${\dot{N}}_{\omega }(\omega ,T,\mu )=\frac{\omega^{2}}{4\pi^{2}c^{2}}f_{\mathrm{BE}}(\omega ,T,\mu )$$

A net number of photons are radiated by the EL diode. In contrast, the NEL diode accepts a net number of photons. The net emitted photon flux for an EL diode is calculated by:

(20) $${\dot{N}}_{\mathrm{net},\mathrm{EL}}=\int_{\omega_{g}}^{\omega_{\mathrm{cut},\mathrm{EL}}}\left[{\dot{N}}_{\omega }\left(\omega ,T_{C},\mu \right)-{\dot{N}}_{\omega }\left(\omega ,T_{H},0\right)\right]d\omega$$

The net absorbed photon flux for an NEL diode is calculated by:

(21) $${\dot{N}}_{\mathrm{net},\mathrm{NEL}}=\int_{\omega_{g}}^{\omega_{\mathrm{cut},\mathrm{NEL}}}\left[{\dot{N}}_{\omega }\left(\omega ,T_{C},0\right)-{\dot{N}}_{\omega }\left(\omega ,T_{H},\mu \right)\right]d\omega$$

The emission or absorption of a photon is associated with the annihilation or creation of an electron-hole pair. The photocurrent density (in the direction of bias) equals the product of the net photon flux and the elementary charge. According to the detailed balance model without considering losses, the current and power required (per unit area) are, respectively:

(22) $$J=q{\dot{N}}_{\mathrm{net}}$$

and

(23) $${\dot{W}}_{\mathrm{db}}=J\left|V\right|=\left|\mu \right|{\dot{N}}_{\mathrm{net}}$$

Here, the subscript “db” refers to the detailed balance analysis, and the relation *µ* = *qV* has been applied to derive Equation (23). The absolute value of *V* or *µ* has been used to ensure that the input work is a positive quantity for both types of systems. Now, the cooling load for the EL refrigeration can be calculated by:

(24) $${\dot{Q}}_{\mathrm{in},\mathrm{db},\mathrm{EL}}={\dot{E}}_{\mathrm{net},\mathrm{EL}}-{\dot{W}}_{\mathrm{db},\mathrm{EL}}$$

For the NEL system, the cooling load is the same as that given in Equation (16) or the net photon energy flux absorbed by the diode. Hence, ${\dot{Q}}_{\mathrm{in},\mathrm{db},\mathrm{NEL}}={\dot{Q}}_{\mathrm{in},2\mathrm{nd},\mathrm{NEL}}$. The coefficient of performance for the EL and NEL systems can be defined as follows:

(25) $$COP_{\mathrm{db},\mathrm{EL}}=\frac{{\dot{Q}}_{\mathrm{in},\mathrm{db},\mathrm{EL}}}{{\dot{W}}_{\mathrm{db},\mathrm{EL}}}=\frac{{\dot{E}}_{\mathrm{net},\mathrm{EL}}-{\dot{W}}_{\mathrm{db},\mathrm{EL}}}{{\dot{W}}_{\mathrm{db},\mathrm{EL}}}$$

and

(26) $$COP_{\mathrm{db},\mathrm{NEL}}=\frac{{\dot{E}}_{\mathrm{net},\mathrm{NEL}}}{{\dot{W}}_{\mathrm{db},\mathrm{NEL}}}$$

Since the diodes are assumed to be ideal, without any defects, and free of any nonradiative losses, this model is expected to keep the process inside the diode to be reversible. However, the results from the detailed balance model and second-law analysis are, generally, not the same. As mentioned in a recent analysis of thermoradiative power generation system [36], the radiative emission and absorption processes at the diode surface may cause additional entropy generation that is implicitly included in the detailed balance model. Entropy generation at the surface due to radiative emission and absorption processes is well known and must be analyzed considering the system as a whole [29]. In the following, the results from both second-law analysis and the detailed balance models are compared for the EL and NEL systems.

## 3. Results and Discussion

### 3.1. EL Refrigeration

**Figure 2:**
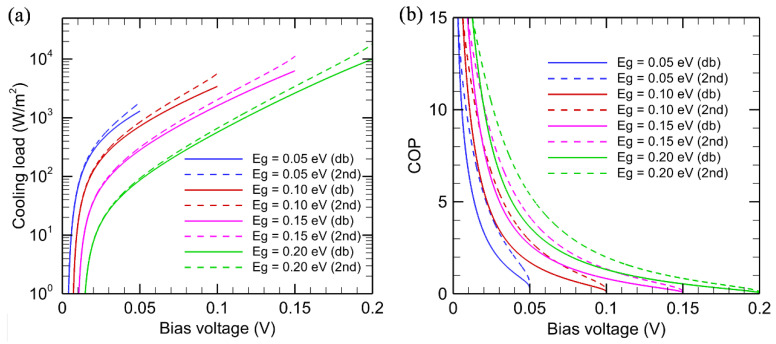
Comparison of (**a**) the cooling load in logarithmic scale and (**b**) coefficient performance of the EL refrigeration system using detailed balance (solid lines) and entropy analysis models (dotted lines), where the diode temperature is set to 300 K and the receiver temperature is set to 320 K.

The performance of EL refrigeration systems for various bandgaps using entropy analysis and detailed balance analysis are plotted in Figure 2. The temperatures of the EL diode and the receiver are assumed to be at 300 K and 320 K, respectively. The predicted results from these two models show similar trends with bias voltage. However, for a given bandgap, the cooling load and *COP* predicted by entropy analysis are consistently greater than those predicted by the detailed balance model. As shown in Figure 2a, the cooling load of the EL system increases as the bias voltage increases. The reason is that the emitted energy flux ${\dot{E}}_{\mathrm{rad}}$ increases quickly as the photon chemical potential increases. It should be noted that the bias voltage cannot exceed *E*_g_/*q* since the photon chemical potential is equal to the quasi-Fermi level split and cannot exceed the semiconductor bandgap. From the detailed balance point of view, for each net emitted photon of energy ℏω, the amount of electric energy that needs to be provided is equal to μ, and the net radiative energy leaving the diode is ℏω−μ. Therefore, the COP will reduce as μ (= *qV*) increases, as shown in Figure 2b.

For the same bias voltage, a larger bandgap reduces the cooling load. However, a larger bandgap allows a larger bias voltage to be applied. Therefore, the maximum cooling load increases with increasing bandgap. This is achieved when the bias voltage is very close to the bandgap. According to the detailed balance model, the cooling load for a semiconductor with *E*_g_ = 0.20 eV is more than 10^4^ W/m^2^ when *µ* → 0.20 eV, and that for a semiconductor with *E*_g_ = 0.05 eV is approximately 1300 W/m^2^ when *µ* → 0.05 eV. However, the *COP* becomes very small when *µ* → *E*_g_. Thus, a trade-off between the cooling load and *COP* must be made. Very high *COP* could only be achieved when the cooling load is very small.

The detailed balance model consistently predicts lower cooling load and *COP* than entropy analysis. By applying Equation (12) to the detailed balance model, it can be shown that:

(27) $${\dot{\sigma }}_{C,\mathrm{db},\mathrm{EL}}={\dot{S}}_{\mathrm{net},\mathrm{EL}}-{\dot{S}}_{\mathrm{in},\mathrm{db}}=\frac{{\dot{Q}}_{\mathrm{in},2\mathrm{nd},\mathrm{EL}}-{\dot{Q}}_{\mathrm{in},\mathrm{db},\mathrm{EL}}}{T_{C}}>0$$

Equation (13) has been used in deriving Equation (27). The additional entropy generation in Equation (27) is due to the entropy generation at the emitting surface [29], implicitly included in the detailed balance model. Since ${\dot{Q}}_{\mathrm{out},2\mathrm{nd},\mathrm{EL}}={\dot{Q}}_{\mathrm{out},\mathrm{db},\mathrm{EL}}$, a larger cooling load in the second-law analysis also gives a larger *COP* than that predicted by the detailed balance model. While the endoreversible formulation sets up a second-law performance limit, it is challenging to remove the surface entropy generation due to radiative emission and absorption processes at the diode surface.

**Figure 3:**
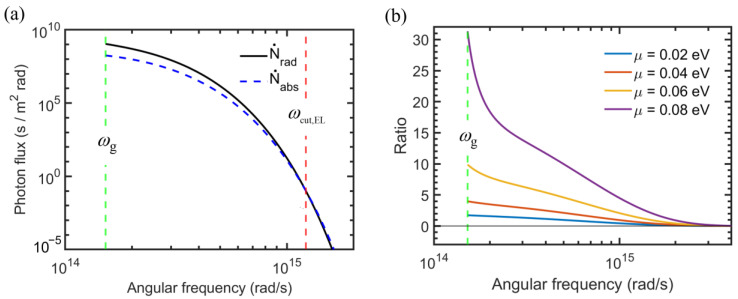
Effect of photon chemical potential on the spectral photon flux for an EL diode with a bandgap of 0.1 eV. (**a**) Spectra of emitted photon flux with *μ* = 0.05 eV and absorbed photon flux. (**b**) Ratio of emitted to absorbed photon fluxes for different photon chemical potentials.

The effect of photon chemical potential may be further illustrated by plotting the emitted and absorbed photon fluxes, as shown in Figure 3a, where the emitter and receiver temperatures are the same as for Figure 2. The bandgap is set to 0.1 eV, corresponding to *ω*_g_ = 1.52 × 10^14^ rad/s, and the photon chemical potential is set at *μ* = 0.05 eV. It can be seen that the emitted photon flux is greater than the absorbed photon flux up to the cut-off frequency *ω*_cut,EL_, which is calculated to be 1.22 × 10^15^ rad/s, according to Equation (6). At $\omega >\omega_{\mathrm{cut},\mathrm{EL}}$, the absorbed photon flux exceeds the emitted photon flux. Note that the logarithmic scale is used for both the abscissa and ordinate of Figure 3a. It can be seen that the spectral photon flux (per unit frequency interval) drops sharply with increasing *ω*. For a blackbody radiator, the emitted energy is less than 1% at photon energies exceeding $10k_{B}T$. At *T* = 300 K, this corresponds to a photon energy of 0.26 eV or angular frequency of 3.9 × 10^14^ rad/s. Hence, in the case of *ω*_g_ = 0.1 eV and *μ* = 0.05 eV, the photon energy beyond $\omega_{\mathrm{cut},\mathrm{EL}}$ is negligibly small. The effect of photon chemical potential is further explored in Figure 3b, which plots the ratio of the emitted photon flux to the absorbed photon flux. It can be seen that the emitted photon flux increases sharply as *μ* increases. When *μ* is very large (e.g., close to the bandgap energy), the net photon flux is dominated by emission from the diode. Note that the cut-off frequency corresponds to the location where the ratio between emitted and absorbed photon fluxes is equal to one and increases with increasing photon chemical potential. The value of *ω*_cut,EL_ is 4.86 × 10^14^ rad/s, 9.72 × 10^14^ rad/s, 1.45 × 10^15^ rad/s, and 1.94 × 10^15^ rad/s for photon chemical potential *µ* = 0.02 eV, 0.04 eV, 0.06 eV, and 0.08 eV, respectively.

### 3.2. NEL Refrigeration

**Figure 4:**
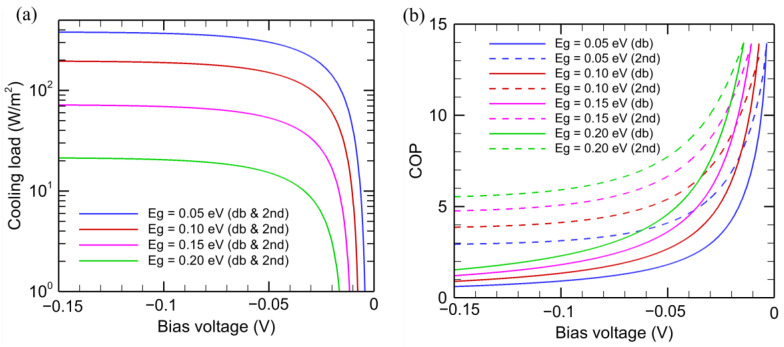
Comparison of the (**a**) cooling load and (**b**) coefficient performance of NEL refrigeration system using detailed balance (solid lines) and entropy analysis models (dotted lines). Only solid lines are shown in the cooling load plot as both detailed balance and entropy analysis predict the same results. The models are calculated considering the NEL diode temperature to be 320 K and the target object to be 300 K.

As for the negative electroluminescent refrigeration system, the cooling load and *COP* are plotted in Figure 4 against the applied bias voltage, which is negative. The NEL diode is maintained at *T*_H_ = 320 K to create a refrigeration effect by absorbing photon energy from a target object maintained at *T*_C_ = 300 K. Note that the cooling load for both the detailed balance and second-law analyses is the same, i.e., ${\dot{Q}}_{\mathrm{in},\mathrm{db},\mathrm{NEL}}={\dot{Q}}_{\mathrm{in},2\mathrm{nd},\mathrm{NEL}}$, as mentioned previously. It can be seen from Figure 4a that the cooling load decreases as the bandgap of the semiconductor increases. This is because the net absorbed energy increases as *ω*_g_ is reduced, since it will reduce the lower bound of the integration for the energy flux. For a given *E*_g_, as the magnitude of voltage increases, the cooling load initially increases, but it gradually saturates to a maximum value. Note that the absorbed photon flux is fixed by *T*_C_ and is independent of the bandgap or applied voltage. An increase in the magnitude of the bias results in a decrease in the emitted photon flux, causing a larger cooling effect. However, for large values of |*μ*|, the emitted photon flux is much smaller than the absorbed photon flux. Hence, further increasing |*V*| does not increase the cooling load by much. Compared with EL refrigeration, the cooling load for NEL is rather limited. For example, the maximum cooling load for the NEL is approximately 380 W/m^2^ with *E*_g_ = 0.05 eV and 200 W/m^2^ with *E*_g_ = 0.1 eV.

The magnitude of the bias voltage must exceed |*V*_th,NEL_| given in Equation (7) to produce any cooling effect. While the *COP* is quite large (exceeding 10) for smaller |*V*|, the cooling power is very small when *V* is close to *V*_th,NEL_. As shown in Figure 4b, the *COP* always decreases as |*V*| increases. From the detailed balance model, each net absorbed photon requires an electric power of |*μ*| to drive the NEL diode and the *COP* is scaled to ℏω/μ, where ω is on the order of $\omega_{g}$. The second-law analysis does not impose such a requirement and predicted a much higher *COP* compared to those obtained from the detailed balance analysis at large values of |*V*|. It can be shown that there exists a finite entropy generation rate in the diode according to

(28) $${\dot{\sigma }}_{H,\mathrm{db},\mathrm{NEL}}={\dot{S}}_{\mathrm{out},\mathrm{db},\mathrm{NEL}}-{\dot{S}}_{\mathrm{net},\mathrm{NEL}}=\frac{{\dot{Q}}_{\mathrm{out},\mathrm{db},\mathrm{NEL}}-{\dot{Q}}_{\mathrm{out},2\mathrm{nd},\mathrm{NEL}}}{T_{H}}>0$$

The above equation can also be written in terms of the power input as follows:

(29) $${\dot{\sigma }}_{H,\mathrm{db},\mathrm{NEL}}=\frac{{\dot{W}}_{\mathrm{db},\mathrm{NEL}}-{\dot{W}}_{2\mathrm{nd},\mathrm{NEL}}}{T_{H}}>0$$

The same argument for the EL refrigeration system can be used here to explain the irreversibility in the detailed balance model since it does not exclude surface entropy generation due to the radiative emission and absorption processes. Therefore, endoreversible thermodynamic analysis predicts a higher *COP*, especially for large magnitudes of bias.

**Figure 5:**
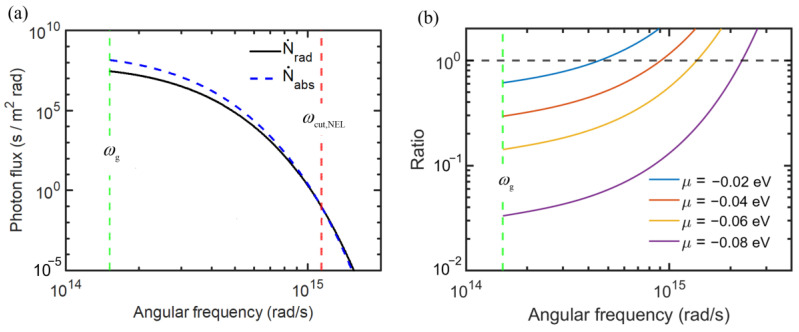
Spectral photon flux for an NEL diode with a bandgap of 0.1 eV at 320 K against a blackbody emitter at 300 K. (**a**) The absorbed and emitted spectral photon flux with a photon chemical potential of −0.05 eV. (**b**) The ratio of the emitted to the absorbed photon flux for different photon chemical potentials.

The spectral photon flux due to absorption and emission from a NEL diode at 320 K and a blackbody at 300 K is shown in Figure 5a, with *E*_g_ = 0.1 eV and *µ* = −0.05 eV. There is a net absorbed photon flux for $\omega_{g}<\omega <\omega_{\mathrm{cut},\mathrm{NEL}}$, even though the diode is at a higher temperature. Note that $\omega_{\mathrm{cut},\mathrm{NEL}}=1.14\times 10^{15}\;\mathrm{rad}/s$ in this case. This is the key to the NEL phenomenon. The spectral photon flux drops very quickly towards higher frequencies and becomes negligible even before the cut-off frequency, as given in Equation (6). Note that the absorbed photon flux is the same as that emitted from a 300 K blackbody and does not change with the diode characteristics. The ratio of emitted to absorbed photon flux is plotted in Figure 5b for several photon chemical potential values. Note that the intersection of these curves with the dashed horizontal line (ratio = 1) corresponds to the cut-off frequency. Increasing |*µ*| results in a significant reduction of the emitted photons. When μ=−0.1 eV, the emitted photon flux is less than 5% of the absorbed photon flux for $\omega <3.9\times 10^{14}\;\mathrm{rad}/s$. Therefore, it becomes clear that further increasing the magnitude of bias would not result in much improvement of the cooling load but will result in a reduction of *COP*, as discussed earlier.

### 3.3. Influence of the Temperature Difference

**Figure 6:**
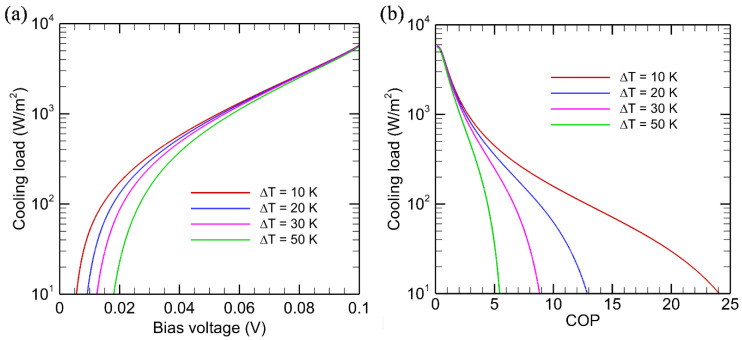
Effect of temperature on the cooling load and *COP* for EL systems based on endoreversible thermodynamics. (**a**) Cooling load versus bias voltage; (**b**) Cooling load versus *COP*. The EL diode with *E*_g_ = 0.1 eV is kept at *T*_C_ = 300 K and the receiver temperature is *T*_H_ = 300 K + Δ*T*.

The effect of temperature difference on the performance of EL and NEL refrigeration is examined in this section. The bandgap of the diode is chosen to be 0.1 eV, corresponding to the range of narrowband mercury-cadmium-telluride (MCT) materials [39]. Only results from the second-law analysis are presented in this section. The cooling load for several temperature differences is shown in Figure 6a for a fixed diode temperature of *T*_C_ = 300 K. The receiver temperature is set to *T*_H_ = *T*_C_ + Δ*T*. As the bias voltage increases, the cooling load increases sharply, but a larger Δ*T* yields a smaller cooling load. Furthermore, to obtain cooling, the minimum bias voltage *V*_th,EL_ also increases. Interestingly, the curves for different Δ*T* values merge as *V* further increases. This can be understood from Figure 3b, which shows that, as *µ* increases, the emitted photon flux overwhelms the absorbed photon flux. A slight increase in *T*_H_ does not affect the cooling load and *COP* much. The cooling load is plotted against the *COP* in Figure 6b. For a high cooling load, the *COP* is a weak function of Δ*T*. However, for a cooling load below 1000 W/m^2^, the *COP* increases sharply as Δ*T* is reduced.

**Figure 7:**
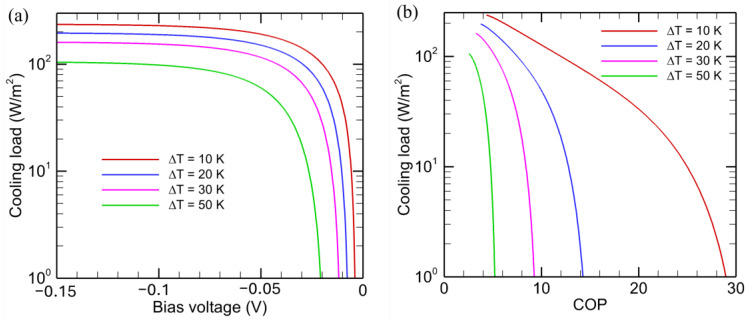
Effect of temperature on the cooling load and *COP* for NEL systems based on endoreversible thermodynamics. (**a**) Cooling load versus bias voltage; (**b**) Cooling load versus *COP*. The NEL diode with *E*_g_ = 0.1 eV is kept at *T*_H_ = 320 K and the receiver temperature is *T*_C_ = 320 K − Δ*T*.

The effect of temperature difference (Δ*T*) on the cooling load for the NEL system is shown in Figure 7a for a diode temperature *T*_H_ = 320 K and a bandgap of 0.1 eV. The emitter temperature is then *T*_C_ = *T*_H_ − Δ*T*. Evidently, the cooling load is higher for a smaller temperature difference and is almost independent of *V* when *V* < −0.1 V. A smaller temperature difference implies a higher target temperature, and the NEL diode can absorb more radiative energy from it. As shown in Figure 7b, the *COP* is much higher for the same cooling load with a smaller temperature difference. While the cooling load for an NEL system is the same with both detailed balance and second-law analysis, the second-law analysis results shown in Figure 7b are much larger than those predicted by the detailed balance; this was shown previously in Figure 4b.

In the analysis presented so far, the diodes are assumed to behave ideally. However, in practice, the diodes experience various loss mechanisms, which are particularly crucial for the EL refrigeration devices. Sub-bandgap radiation gives rise to a net thermal radiative transfer from the hot surface to the cold surface and, thus, reduces the cooling performance. Spectrally selective emitters or absorbers may be necessary to suppress subwavelength radiation. At low bias voltages, Shockley-Read-Hall recombination/generation is a dominant nonradiative mechanism, while at high bias voltages, Auger recombination process may play a significant role [2,27]. These mechanisms reduce the internal quantum efficiency of the diode. To increase the emissivity and reduce surface reflection, antireflection coatings may be desired in practice. Photonic crystals have been considered to improve the light extraction efficiency [40]. The product of the internal quantum efficiency and light extraction efficiency is the external quantum efficiency of the EL diode. For effective cooling to occur, the external quantum efficiency must exceed the ratio of the photon chemical potential to the bandgap energy [2]. In the case of NEL devices, the emitter is passively cooled and not connected to an electrical power source. Therefore, high internal quantum efficiency is not a strict requirement. However, internally efficient diodes are still preferred as nonidealities raise the temperature of the NEL diode via joule heating [19] and, thus, diminish the cooling performance of the system.

## 4. Conclusions

This study establishes the fundamental thermodynamic limit of the cooling performance of the electroluminescent refrigeration systems using second-law or entropy analysis. The diodes are considered to be ideal and the endoreversible assumption is adopted. The cooling load and coefficient of performance determined using this method are compared with those of the detailed balance model. The entropy analysis consistently puts forth better refrigeration load and *COP* because the detailed balance analysis implicitly presumes that entropy is generated on the diode surface. Additionally, the effect of temperature difference between the emitter and receiver of the refrigeration mechanisms is investigated using entropy analysis. The methodology and the analysis shown in this study provide the theoretical benchmark for the cooling performance that can be achieved in EL and NEL systems. Currently, there is still a lack of experimental demonstrations of EL and NEL systems due to the difficulties associated with nonradiative processes, other nonidealities, and sub-bandgap radiation losses. The radiative heat flux can be significantly enhanced in the near field, resulting in higher cooling capacities. A detailed analysis of entropy generation in near-field electroluminescent devices is underway to understand the impact of irreversible processes on the performance of such devices.

## Data Availability

All data is presented in graphs. The source of the data is available upon reasonable requests to the corresponding author.

## Nomenclature

c	speed of light in a vacuum, m/s
$E_{g}$	bandgap energy, J
${\dot{E}}_{\mathrm{abs}}$	rate of photon energy absorbed by the diodes, W/m^2^
${\dot{E}}_{\mathrm{net},\mathrm{EL}}$	rate of net energy flow by radiation from the EL diode to the receiver, W/m^2^
${\dot{E}}_{\mathrm{net},\mathrm{NEL}}$	rate of net energy absorbed by radiation by the NEL diode from the target, W/m^2^
${\dot{E}}_{\mathrm{rad}}$	rate of photon energy emitted by the diodes, W/m^2^
${\dot{E}}_{\omega }$	spectral emissive power, J/m^2^·rad
$f_{\mathrm{BE}}$	modified Bose-Einstein distribution
ℏ	reduced Planck constant, 1.055 × 10^−34^ J·s
$k_{B}$	Boltzmann constant, 1.381 × 10^−23^ J/K
${\dot{N}}_{\mathrm{net},\mathrm{EL}}$	net photon flux from the EL diode to the receiver, m^−2^
${\dot{N}}_{\mathrm{net},\mathrm{NEL}}$	net photon flux to the NEL diode from the target object, m^−2^
${\dot{N}}_{\omega }$	spectral photon flux, s/m^2^·rad
${\dot{Q}}_{\mathrm{in}}$	rate of heat transferred from the cold reservoir, W/m^2^
${\dot{Q}}_{\mathrm{out}}$	rate of heat transferred out to the hot reservoir, W/m^2^
q	elementary charge, 1.602 × 10^−19^ C
${\dot{S}}_{\mathrm{in}}$	rate of entropy transferred from the cold reservoir, W/K·m^2^
${\dot{S}}_{\mathrm{abs}}$	rate of photon entropy absorbed by the diodes, W/K·m^2^
${\dot{S}}_{\mathrm{net},\mathrm{EL}}$	rate of photon entropy transferred from the EL diode to the receiver, W/K·m^2^
${\dot{S}}_{\mathrm{net},\mathrm{NEL}}$	rate of photon entropy absorbed by the NEL diode from the target object, W/K·m^2^
${\dot{S}}_{\mathrm{out}}$	rate of entropy transferred to the hot reservoir, W/K·m^2^
${\dot{S}}_{\mathrm{rad}}$	rate of photon entropy emitted by the diodes, W/K·m^2^
${\dot{S}}_{\omega }$	spectral entropy flux, J/K·m^2^·rad
*T*	absolute temperature, K
*V*	bias voltage of the cell, V
*V*_th_	threshold bias voltage, V
$\dot{W}$	power absorbed by the refrigeration system, W/m^2^
Greek symbols	
*μ*	photon chemical potential, J
$\dot{\sigma }$	entropy generation rate, W/K·m^2^
*ω*	angular frequency, rad/s
Subscripts	
2nd	second-law analysis
db	detailed balance analysis
C or H	cold or hot reservoir
cut	cut-off
g	bandgap
List of abbreviations	
*COP*	coefficient of performance
EL	electroluminescent cooling
LED	light emitting diode
MCT	mercury-cadmium-telluride
NEL	negative electroluminescent cooling
